# Incidence and risk factors for febrile neutropenia of patients with diffuse large B-cell lymphoma receiving R-CHOP-21 in China

**DOI:** 10.1007/s00520-023-08250-z

**Published:** 2023-12-19

**Authors:** Wenshuai Zheng, Zhaoguang Chen, Shibin Zhu, Longcan Cheng, Yalei Hu, Yuhui Yang, Min Tan, Hongmei Ning, Lixun Guan

**Affiliations:** 1 Department of Hematology, Hainan Hospital of Chinese PLA General Hospital, Sanya, 572000 Hainan China; 2 Department of Critical Care Medicine, Hainan Hospital of Chinese PLA General Hospital, Sanya, 572000 Hainan China; 3 Department of Laboratory Medicine, Hainan Hospital of Chinese PLA General Hospital, Sanya, 572000 Hainan China; 4 Senior Department of Hematology, Fifth Medical Center of Chinese PLA General Hospital, Beijing, 100071 China

**Keywords:** Febrile neutropenia, Diffuse large B-cell lymphoma, R-CHOP-21, Risk factors, Granulocyte colony–stimulating factors, Prophylaxis, Risk score system

## Abstract

**Objective:**

Febrile neutropenia (FN) is a serious complication of patients with diffuse large B-cell lymphoma (DLBCL) receiving R-CHOP-21. The prophylactic use of granulocyte colony–stimulating factors (G-CSFs) can significantly reduce the risk of FN. International guidelines recommend G-CSFs for patients receiving chemotherapy with FN risk of 20% or 10 to 20% with defined risk factors. However, there are few studies on the incidence and risk factors of FN in patients with DLBCL receiving R-CHOP-21, especially in patients without primary G-CSF prophylaxis.

**Methods:**

We conducted a retrospective analysis for the clinical data of 103 patients with DLBCL who underwent first R-CHOP-21 without primary G-CSF prophylaxis. The objective of the assessment was the incidence and risk factors of FN after the first chemotherapy cycle.

**Results:**

After the first chemotherapy cycle, the incidence of FN was 20.4%. Multivariate analysis showed that age ≥ 65 years, bone marrow involvement, albumin < 35 g/L, and average relative dose intensity ≥ 80% were independent risk factors for FN. According to risk factors, we created a risk score system. The incidence of FN in the low-, intermediate- and high-risk groups was 5.6%, 17.2%, and 61.9%, respectively.

**Conclusion:**

Our data indicated that R-CHOP-21 itself is associated with a high-risk regiment for FN. We recommend that intermediate/high-risk patients should actively consider primary G-CSF prophylaxis to reduce the incidence of FN after chemotherapy.

## Introduction

Diffuse large B-cell lymphoma (DLBCL) is the most common lymphoma subtype [1]. Currently, a 21-day cycle of chemotherapy regimen comprising rituximab, cyclophosphamide, doxorubicin, vincristine, and prednisolone (R-CHOP-21) is the standard treatment for DLBCL [2, 3]. Febrile neutropenia (FN) is one of the major adverse reactions after chemotherapy, which often result in prolonged hospitalizations and increased treatment costs. Otherwise, the development of FN can lead to unwanted delay or reduction of chemotherapy dose, which may impact curative effect [4, 5]. So, the management and prevention of FN is important for patients with DLBCL. It has been proven that primary granulocyte colony–stimulating factors (G-CSFs) prophylaxis can significantly reduce the incidence of FN induced by chemotherapy [6–8]. But the high cost of polyethylene glycol (PEG)-G-CSFs and the inconvenience of short-acting G-CSFs have prevented it from being routinely used in all patients receiving myelosuppressive chemotherapy.

According to the incidence of FN, chemotherapy regimens are categorized into high- (20%), intermediate- (10–20%), or low (< 10%)-risk for FN [8]. The guidelines from the National Comprehensive Cancer Network (NCCN) recommend primary G-CSF prophylaxis for patients receiving high-risk chemotherapy regiments, and prophylaxis is not recommended for most patients receiving low-risk and intermediate-risk chemotherapy regiments without identified risk factors [9]. However, an invasive model based on average population risks and chemotherapy regimens may not be the most effective way to determine the primary G-CSF prophylaxis. In addition to chemotherapy regimens, other factors, such as age, complications, disease stage, physical status, and laboratory values, also should be considered, especially for patients receiving intermediate-risk chemotherapy regimens. In most guidelines, R-CHOP-21 is classified as an intermediate-risk chemotherapy regimen [9–11]. Therefore, it is important to recognize what the high-risk features are of FN in patients with DLBCL treated with R-CHOP-21 to determine the primary G-CSF prophylaxis. However, few studies have investigated this question, and many patients in these studies had already received primary G-CSF prophylaxis, which cannot reflect the real incidence and risk factors of FN [12–16]. In addition, there have been no reports on the incidence and risk factors of FN in patients with DLBCL who underwent first R-CHOP-21 without primary G-CSF prophylaxis in China. So, we performed this study to provide reference for the primary G-CSF prophylaxis in patients with DLBCL.

## Methods

### Patients and study design

Between January 1, 2016, and June 30, 2022, patients who were newly diagnosed with DLBCL [1], aged ≥ 18 years, and completed at least one cycle of R-CHOP-21 were enrolled in this study. Patients with HIV positivity, central nervous system involvement, or incomplete inpatient and outpatient follow-up data were excluded. All patients received R-CHOP-21 (rituximab 375 mg.m^−2^ on day 0, cyclophosphamide 750 mg.m^−2^ on day 1, doxorubicin 50 mg.m^−2^ or pegylated liposomal doxorubicin 25 mg.m^−2^ on day 1, vincristine 1.4 mg.m^−2^ [up to a maximum dose of 2 mg] on day 1, and prednisolone 60 mg.m^−2^.day^−1^ on days 1 to 5). Most of the patients received the standard dose of chemotherapy, but the actual dose of elderly patients and patients with complications or poor organ function was appropriately reduced by the attending physician. After chemotherapy, all patients did not receive the primary G-CSF prophylaxis, and most of patients were discharged. Blood routine examination and hepatorenal function were examined in the outpatient department at 3, 6, 9, and 12 days. Patients with FN were given a microbial culture, symptomatic anti-infection treatment, and G-CSFs (filgrastim, 5 µg/kg 1/day) until ANC > 0.5 × 10^9^/L. Data collected at baseline included age, sex, disease stage, bone marrow involvement, extranodal involvement, Eastern Cooperative Oncology Group performance status (ECOG PS), average relative dose intensity (ARDI), and laboratory values (lactate dehydrogenase [LDH], albumin, absolute neutrophil count [ANC], hemoglobin, and platelet). According to risk factors of FN, we create a risk score system which is an ordered categorical variable categorized as low-, intermediate-, and high-risk. All methods used in this study were carried out in accordance with the Helsinki Declaration. This is a retrospective, anonymous analysis of clinical and epidemiological data only; thus, an ethical approval was not required.

### Definitions and evaluations

The primary endpoint was the incidence of FN after the first cycle of chemotherapy. FN was defined as a single oral temperature ≥ 38.3 ℃ (axillary temperature ≥ 38.0 ℃) or oral temperature ≥ 38.0 ℃ (axillary temperature ≥ 37.7 ℃) lasting more than 1 h and neutropenia with an ANC of < 0.5 × 10^9^/L or an ANC that is expected to decrease to < 0.5 × 10^9^/L after 48 h [17]. The secondary endpoint was the risk factors of FN after the first cycle of chemotherapy. The relative dose intensity (RDI) was defined as the proportion of the dose actually delivered to the standard dose. The average relative dose intensity (ARDI) was calculated by averaging the delivered RDI of cyclophosphamide and doxorubicin because other drugs have a mild effect on bone marrow suppression.

### Statistical analysis

Baseline demographics and clinical characteristics were summarized as mean ± standard deviation for continuous variables and as percentages for categorical variables. The univariate analysis of factors involved in the development of FN after the first cycle of chemotherapy was conducted by the chi-square test. Based on outcomes of univariate analysis, a stepwise logistic regression model was constructed by combining the statistically relevant indicators (with *p* < 0.10 in the univariate model) or clinically significant indicators. All tests were two-sided, and *p* < 0.05 was considered significant. All statistical analyses were conducted by SPSS 23.0.

## Results

### Patient characteristics

Finally, a total of 103 consecutive eligible patients were enrolled in this study; the baseline demographics and clinical characteristics are summarized in Table 1. Of the 103 patients, 54 were men (52.4%). Patients had a mean (SD) age of 56 (15) years. Slightly over half of the patients (57, 55.3%) received pegylated liposomal doxorubicin. Most patients (76, 73.8%) had an ECOG PS of 0–1. Most patients (75, 72.8%) had an advanced disease (Ann Arbor stage III–IV). Most patients (72, 69.9%) had a number of extranodal involvement ≤ 1. Most patients (66, 64.1%) had an ARDI ≥ 80%. About 30% of patients had bone marrow involvement.

**Table 1:** Demographic and clinical characteristics of patients

	*N* = 103
Age (years)	56 (± 15)
Sex	
Male	54 (52.4%)
Female	49 (47.6%)
Therapy	
Doxorubicin	46 (44.7%)
Pegylated liposomal doxorubicin	57 (55.3%)
ECOG PS	
0–1	76 (73.8%)
2–4	27 (26.2%)
Ann Arbor stage	
I–II	28 (27.2%)
III–IV	75 (72.8%)
Lactate dehydrogenase (U/L)	381 (± 526)
Number of extranodal involvement	
≤ 1	72 (69.9%)
> 1	31 (30.1%)
Bone marrow involvement	
Yes	33 (32.0%)
No	70 (68.0%)
Absolute neutrophil count (× 10^9^/L)	4 (± 2.6)
Hemoglobin (g/L)	117 (± 25)
Albumin (g/L)	37.4 (± 5.6)
Average relative dose intensity	
< 80%	37 (35.9%)
≥ 80%	66 (64.1%)

Data are presented as *n* (%) or mean ± SD

*ECOG PS* Eastern Cooperative Oncology Group performance status

### Univariate and Multivariate Analysis of Risk Factors Associated with FN

In the univariate analysis, the incidence of FN was significantly higher in patients with ANC < 2.0 × 10^9^/L vs ≥ 2.0 × 10^9^/L (*p* = 0.009), those with hemoglobin < 120 g/L vs ≥ 120 g/L (*p* = 0.003), those with albumin < 35 g/L vs ≥ 35 g/L (*p* = 0.002), those who had bone marrow involvement vs those who did not have bone marrow involvement (*p* < 0.001), and those who received ARDI ≥ 80% vs those who received ARDI < 80% (*p* = 0.021). There were no differences in the incidence of FN by age, sex, therapy, ECOG PS, Ann Arbor stage, LDH, number of extranodal involvement, and platelet (Table 2). The statistically and clinically relevant factors were included in the multivariate logistic regression model. As a result, age ≥ 65 (odds ratio [OR], 4.014; 95% confidence interval [CI], 1.074–15.002, *p* = 0.039), bone marrow involvement (OR, 5.276; 95% CI, 1.618–17.210, *p* = 0.006), albumin < 35 g/L (OR, 4.234; 95% CI, 1.246–14.381, *p* = 0.021), and ARDI ≥ 80% (OR, 7.015; 95% CI, 1.401–35.117, *p* = 0.018) were found to be independent risk factors for FN (Table 3).

**Table 2:** Univariate analysis of risk factors for febrile neutropenia

Characteristics	*N* = 103 *n*/*N* (%)	Chi-square (*p* value)
Age (years)		
< 65	11/68 (16.2%)	2.187
≥ 65	10/35 (28.6%)	(0.139)
Sex		
Male	10/54 (18.5%)	0.245
Female	11/49 (22.4%)	(0.621)
Therapy		
Doxorubicin	11/46 (23.9%)	0.087
Pegylated liposomal doxorubicin	10/57 (17.5%)	(0.768)
ECOG PS		
0–1	14/76 (18.4%)	0.691
2–4	7/27 (25.9%)	(0.406)
Ann Arbor stage		
I–II	5/28 (17.9%)	0.152
III–IV	16/75 (21.3%)	(0.697)
Lactate dehydrogenase		
≤ 250 U/L	7/53 (13.2%)	3.468
> 250 U/L	14/50 (28.0%)	(0.063)
Number of extranodal involvement		
≤ 1	12/72 (16.7%)	2.041
> 1	9/31 (29.0%)	(0.153)
Bone marrow involvement		
Yes	15/32 (46.9%)	18.796
No	6/70 (8.6%)	(< 0.001)
Absolute neutrophil count		
< 2.0 × 10^9^/L	8/19 (42.1%)	6.769
≥ 2.0 × 10^9^/L	13/84 (15.5%)	(0.009)
Hemoglobin		
< 120 g/L	7/24 (29.2%)	8.602
≥ 120 g/L	14/79 (17.7%)	(0.003)
Platelet		
< 100 × 10^9^/L	7/24 (29.2%)	1.486
≥ 100 × 10^9^/L	14/79 (17.7%)	(0.223)
Albumin		
< 35 g/L	12/30 (40.0%)	10.030
≥ 35 g/L	9/73 (24.3%)	(0.002)
Average relative dose intensity		
< 80%	3/37 (8.1%)	5.365
≥ 80%	18/66 (27.3%)	(0.021)

*ECOG PS* Eastern Cooperative Oncology Group performance status

**Table 3:** Multivariate analysis of risk factors for febrile neutropenia

Characteristics	OR	95% CI	*p* value
Age (years) (≥ 65, < 65)	4.014	1.074–15.002	0.039
Bone marrow involvement (yes, no)	5.276	1.618–17.210	0.006
Albumin (g/L) (< 35, ≥ 35)	4.234	1.246–1 4.381	0.021
Average relative dose intensity (≥ 80%, < 80%)	7.015	1.401–35.117	0.018

*OR* odds ratio, *CI* confidence interval

### Risk score system

The incidence of FN after the first chemotherapy was 20.4%. In order to further identify patients at a higher-risk for FN, we generated a risk score system based on risk factors in the multivariate analysis (albumin < 35 g/L = 1, bone marrow involvement = 1, age ≥ 65 = 1, ARDI ≥ 80% = 1). Scores ≤ 1 were considered the low-risk group, 2 intermediate-risk group, and ≥ 3 high-risk group. The incidence of FN in the low-, intermediate-, and high-risk groups were 5.6% (3/53), 17.2% (5/29), and 61.9% (13/21), respectively.

## Discussion

We examined the incidence and risk factors of FN among patients with DLBCL who received the first R-CHOP-21 without primary G-CSF prophylaxis.

According to current guidelines, chemotherapy regimens with the occurrence rate of FN higher than 20%, 10–20%, and less than 10% are considered high-, intermediate-, and low-risk for FN, respectively [9]. The classification of these chemotherapy regimens was based on several clinical trials and included only qualified patients. As a result, the hematotoxicity is often underestimated in these highly selected patients. In most guidelines, R-CHOP-21 is regarded as an intermediate-risk regimen for FN [9–11]. However, the occurrence rate of FN in several studies of R-CHOP-21 including patients who had received primary G-CSF prophylaxis was 17–22% [18, 19]. In this study, 20.4% of patients without primary G-CSF prophylaxis experienced FN, which suggests that R-CHOP-21 is a high-risk regimen for FN in real clinical situations. Meanwhile, this study showed that the risk of FN was inconsistent among different patients. So, it is important to find patients who have high-risk features related to FN to prophylactically use G-CSFs.

In several studies including non-Hodgkin’s lymphoma (NHL) patients, being older than 60 or 65 years was found to be an independent risk factor for FN [13, 14, 20, 21]. Salar et al. reported that old age and poorer performance status were both independent risk factors for FN in NHL patients treated with R-CHOP-21 [15]. In this study, being older than 65 years was an independent risk factor for FN. This may be related to the worse performance status of elder patients, in which 37.1% (13/35) patients who were older than 65 years had an ECOG score ≥ 2 points, while only 20.6% (14/68) patients who were younger than 65 years had an ECOG score ≥ 2 points.

In this study, among disease-related risk factors including Ann Arbor stage, number of extranodal involvement, and bone marrow involvement, only bone marrow involvement is an independent risk factor for FN. The result is similar to Choi’s study about patients with DLBCL receiving R-CHOP-21 [16]. This may be related to the inhibition of granulocyte hyperplasia caused by bone marrow involvement. The portion of ANC < 2.0 × 10^9^/L in patients with bone marrow involvement (9/33) is higher than in patients without bone marrow involvement (10/70).

Many studies including NHL have found that abnormal laboratory values are important risk factors for FN, for example, lower baseline albumin [6], hemoglobin [14], and ANC [21]. In this study, We analyzed the effect of pretreatment baseline laboratory data on FN, including ANC, hemoglobin, platelet, albumin, and LDH and found that only low baseline albumin (< 35 g/L) was an independent risk factor for FN. Lower levels of albumin may indicate an impaired systemic condition, which can further exacerbate the condition and lead to a higher incidence of FN.

In addition to chemotherapy regimens, we often adjust the dose of chemotherapy according to the different states of patients in clinical practice, such as, we may reduce the dose of cyclophosphamide or doxorubicin to reduce treatment-related complications in older patients or patients with poor organ function. Therefore, the actual strength of chemotherapy may also lead to different incidences of FN. In this study, ARID ≥ 80% is an independent risk factor for FN, which confirmed the above view and is consistent with Lyman’s study about patients with NHL receiving CHOP [21].

This study showed that R-CHOP-21 is a high-risk chemotherapy regimen for FN in patients with DLBCL not receiving primary G-CSF prophylaxis. In order to further identify higher-risk patients, we divided patients into low-, intermediate- and high-risk groups according to risk factors (bone marrow involvement, albumin < 35g/L, age ≥ 65 years, and ARDI ≥ 80%). The incidence of FN in three groups was 5.6%, 17.2%, and 61.9%, respectively. We suggest that primary G-CSF prophylaxis is unnecessary for low-risk patients but should be used for high-risk patients, while G-CSFs should be used prophylactically as far as possible for intermediate-risk patients according to their will and economic situation.

This study has several limitations, such as those inherent to observational and retrospective studies. This study was conducted at a single center which may hamper the generalizability of the results. In addition, because the follow-up of most patients after chemotherapy was performed in the outpatient department, it is possible that the detection of FN was not entirely accurate.

## Conclusions

Our study indicated that the incidence of FN in patients with DLBCL who received the first cycle R-CHOP-21 without primary G-CSF prophylaxis was 20.4%, which means that R-CHOP-21 itself is a high-risk regimen for FN. Additionally, the presence of age ≥ 65 years, bone marrow involvement, albumin < 35 g/L, and ARDI ≥ 80% were independent risk factors for FN. Patients with the above risk factors will have a higher incidence of FN. According to our risk scores system, patients with intermediate/high risk should routinely receive primary G-CSF prophylaxis after the first R-CHOP-21. Of course, a future large-scale randomized study should be performed to further verify the results of this retrospective study.

## Data Availability

Authors have full control of primary data, which can be made available to editors upon request.
